# The Fifteenth International Medical Congress

**Published:** 1904-05-28

**Authors:** 


### The Fifteenth International Medical Congress.

The authorities who are responsible for the conduct of the fifteenth international Congress are making timely arrangements for the next meeting, which is to be held at Lisbon on April 19th to April 26th, 1906. A preliminary programme is issued, "in English as she is spoke" it is true, but with every intention to take time by the forelock and not leave any stone unturned which may perfect the arrangements and prevent the chaos which spoilt the Rome and Madrid meetings. The subscription is fixed at £1 for each member, and the Portuguese Committee has very wisely restricted the membership to doctors and such scientific men as are recommended by the national and general committee of the Congress. In Spain it is notorious that hundreds went to Madrid whose only qualification for membership consisted in a general interest in science. Veterinary surgeons, pharmaceutical chemists and wholesale druggists were all represented, with a result that the authorities found it impossible to cope with the numbers present, the scientific side of the Congress was neglected, and the picnic aspect was spoilt by the excessive demand for tickets. It is to be hoped that the Lisbon Congress will prove more satisfactory, for to be forewarned is to be forearmed, and the limitation of numbers is the first step in a right direction. The work of the executive depends largely upon the zeal of the national committees, for if each works well in its own country good papers are secured and the Congress is a success; but if the national committees are lukewarm little is done and the Congress does not come up to a high standard. The National Committee for Great Britain and Ireland has been recently re-organised. It consists of his Majesty's principal advisers in the navy, army, Indian medical services, a representative from each University and medical corporation of the United Kingdom, the Presidents of some of the chief medical societies, and representatives of general practitioners in England, Scotland, Ireland and Wales. The President is Dr. F. W. Pavy, F.R.S. and the honorary secretaries are Dr. Clive Riviere and Mr. D'Arcy Power. The Portuguese executive committee intend to print all the official reports before the meeting, and it is necessary that papers should be sent to the secretary-general (Dr. Miguel Bombarda, Hospital de Rilhafolles, Lisbon) before December 31, 1905. This should obviate the difficulty felt at Madrid, where the abstracts of papers were often unobtainable until after the communication had been made. The sectional meetings were thus robbed of much of their interest, for it was often impossible to follow the speaker, and no adequate discussion took place in spite of many tempting opportunities. The official language of the Congress is French, but in the general assemblies as well as in the sections English, German, and French will be used indiscriminately. With a fine generosity the Portuguese have waived the use of their own language; in the words of the circular "this has only been done with the intention of diminishing the number of languages spoken: there can be no jalousy (*sic*) when the legislator begins by sacrifising (*sic*) himself." The work of the Congress is divided into 17 sections: anatomy, physiology, general pathology, including bacteriology, therapeutics and pharmacology; medicine, diseases of children, neurology with criminal anthropology, diseases of the skin, including syphilis; surgery, urinary medicine and surgery, ophthalmology, laryngology, obstetrics and diseases of women, hygiene and epidemology, military medicine, legal medicine, colonial and naval medicine. Thus it will be seen that the Congress sweeps all branches of medical science into its net; and if our Portuguese friends will take the trouble to arrange matters in a thoroughly satisfactory manner and make the meeting a success, it will redound to their credit, and will go far to raise the rather mean opinion which the Teutonic races hold of the Latin power of organisation.

